# Association between 24-hour movement behaviors and the frequency of colds in Chinese middle school students: a compositional and isotemporal substitution analysis

**DOI:** 10.7717/peerj.21124

**Published:** 2026-04-24

**Authors:** Huanmi Nie, Ran Liu, Wei Xiang, Lin Zhu, Jinkun Li, Junyu Shen

**Affiliations:** 1 School of Physical Education and Sports, Central China Normal University, Wuhan, Hubei, China; 2 School of Physical Education, Dali University, Yunnan, Dali, China; 3 School of Physical Education, Yan’an University, Yanan, Shaanxi, China

**Keywords:** 24-hour movement behaviors, Compositional data, Isotemporal substitution model, Frequency of colds, Middle school students

## Abstract

**Background:**

Physical activity (PA) exerts a significant impact on global health and has been associated with a reduced risk of common cold. This study aims to examine the correlation between PA and the frequency of colds among middle school students in China.

**Methods:**

A cross-sectional study was conducted among 356 middle school students aged 12–13 in Wuhan, Hubei Province, China. The 24-hour movement behaviors were measured using ActiGraph wGT3X-BT accelerometer, and participants reported the number of the frequency of cold s over the past year. Compositional linear regression and isotemporal substitution models were employed to analyze the association between time allocation to 24-hour movement behaviors and the frequency of colds, and to predict changes in frequency of colds following time reallocation among these behaviors.

**Results:**

(1) The time spent in moderate-to-vigorous physical activity (MVPA) was the most stable component of 24-hour movement behaviors among middle school students. When activity patterns changed, the highest probability of time reallocation occurred with light physical activity (LPA). (2) A significant association was observed between 24-hour movement behaviors and the frequency of colds among middle school students. Specifically, the proportion of MVPA time was negatively correlated with the frequency of colds among middle school students. The proportion of time spent in sleep (SP) was positively associated with the frequency of colds, No significant associations were observed between time allocation in sedentary behavior (SB) or light physical activity (LPA) and the frequency of colds. (3) The 15-minute isotemporal substitution model predicted significant reductions in the frequency of colds when replacing both sedentary behavior (SB) and sleep (SP) with MVPA. Conversely, when MVPA was replaced by either SB or SP, the frequency of colds increased significantly. Notably, the substitution of SP with MVPA demonstrated the most substantial effect in reducing the frequency of colds. (4) The dose-response relationship (ranging from −30 to +30 minutes) revealed asymmetric effects on the frequency of colds when mutually substituting MVPA with either SB or SP.

**Conclusion:**

To reduce the frequency of colds among middle school students in the future, educators and parents should focus on the overall framework of 24-hour movement behaviors, increasing MVPA and reducing SB are effective strategies to achieve this goal.

## Introduction

Physical activity (PA) is a key component of a healthy lifestyle. Insufficient or excessive physical activity may increase the risk of infectious diseases (Tiollier et al., 2005; Nieman, 2010; Lee et al., 2012; Durstine et al., 2013). Among these is the common cold, a prevalent self-limiting upper respiratory tract infection. On average, adults experience two to three episodes annually (Allan & Arroll, 2014), with multiple adverse effects including sleep disturbances, work absenteeism, and decreased productivity (Dicpinigaitis et al., 2015). Regular moderate-to-vigorous physical activity can promote resilience to the common cold by increasing CD4+ T-cell counts and salivary immunoglobulin A (IgA) concentrations, thereby enhancing immune system function (Chastin et al., 2021).

To objectively elucidate the relationship between exercise intensity and the common cold, a growing number of researchers have begun to investigate this association. In a one-year randomized controlled trial targeting postmenopausal women, Chubak et al. (2006) demonstrated that moderate-intensity exercise training for 12 months significantly reduced the incidence of common colds in this population. Tu, Lu & Tao (2022) used self-report questions to measure Chinese adults’ preferences for physical activity (PA) in terms of intensity, frequency, and duration. The study found that the relationship between exercise intensity and the common cold followed a U-shaped dose–response curve, High intensity combined with high frequency was most strongly associated with an increased incidence of colds. Tang et al. (2024) investigated the dose–response relationship between weekly physical activity levels and the frequency of colds among middle-aged and elderly adults in China. The results suggested that regular Moderate and high intensity physical activity may reduce the risk of colds in this population. One other study is relevant here. Matthews et al. (2002) focused on respiratory infections but not colds and found that adults who engaged in regular moderate-to-vigorous physical activity exhibited a 20–30% reduction in annual incidence rate of upper respiratory tract infections.

However, these studies analyzed the association between individual activity behaviors and colds (respiratory infections), neglecting the inherent interconnectedness among various activity behaviors. Prochaska, Spring & Nigg (2008) emphasizes that in health promotion, we should not focus solely on the impact of a specific activity behavior (such as moderate-to-vigorous physical activity) on health. Instead, it is essential to holistically consider the combined effects of multiple behaviors—including low-intensity physical activity, sedentary behavior, and sleep—throughout the entire day. In 2016, Canada first proposed the concept of “24-hour Movement Behaviors” and released 24-hour movement guidelines covering all age groups. Subsequently, Australia, New Zealand, South Africa, Finland, Croatia, and the WHO also successively released their own 24-hour movement guidelines.The introduction of these guidelines marked a paradigm shift in the field of physical activity and health promotion research (Tremblay et al., 2016; Rosenberger et al., 2019). Currently, within the macro research framework of 24-hour movement behaviors, the association between such behaviors and the frequency of colds has not yet been systematically explored. Therefore, investigating the impact of time allocation in 24-hour movement behaviors on cold frequency can provide a basis for effectively guiding individuals in reasonably distributing their daily activities and promoting physical and mental health.

24-hour Movement Behaviors integrates physical activity, sedentary behavior, and sleep throughout the day, forming a complementary health behavior pattern (Tremblay et al., 2016). These activities adhere to the “compositional nature” of time-use data—specifically, the “fixed-sum constraint” property, meaning that increasing one activity necessarily reduces time spent on others to maintain the total daily duration of 24 h (Pedisic, 2014; Pedisic, Dumuid & Olds, 2017). When analyzing such compositional data, traditional statistical methods often fail to accurately reflect the true relationships due to issues like multicollinearity. To address this, scholars have adopted innovative compositional data analysis approaches, particularly isotemporal substitution modeling combined with log-ratio transformation techniques, to precisely assess the health impacts of 24-hour movement behaviors (Chastin et al., 2015; Dumuid et al., 2018; Dumuid et al., 2019). Unlike traditional isotemporal substitution models that primarily focus on the direct health effects of substituting a single behavior (Mekary et al., 2009), compositional and isotemporal substitution analysis enables in-depth point-to-point comparisons to evaluate the health benefits of inter-behavior substitutions (Chastin et al., 2015). Crucially, it accounts for the interdependencies between behaviors within daily activity patterns and their collective impact on overall health. This approach provides a holistic perspective for understanding 24-hour movement behaviors and their comprehensive effects on physical and mental well-being. Currently, research on compositional data analysis in adolescents primarily involves studies on the associations between 24-hour movement behaviors and mental health, obesity, depressive/anxiety symptoms, body mass index (BMI), executive function, and cardiometabolic health in children and adolescents (Bezerra et al., 2020; Fairclough et al., 2021; Gába et al., 2021; De Faria et al., 2022; Marshall et al., 2025). Most of these studies are based on western adolescent populations, while there is a lack of research applying compositional data analysis to explore the comprehensive impact of 24-hour movement behaviors on the physical health of middle school students in China. In China, the cultural values linking academic achievement with future socio-economic status (Hesketh & Ding, 2005), coupled with the high emphasis and investment parents place on their children’s academic performance, result in Chinese adolescents generally bearing a heavier academic burden compared to their counterparts in other countries and regions. Excessive academic burden has been identified as a significant factor affecting physical activity, lifestyle, and sedentary behavior among middle school students (Sun et al., 2013), Concurrently, the widespread sleep deprivation it causes has had a notably negative impact on their physical and mental health development (Owens & Weiss, 2017).

Based on this, this study is the first employ compositional data analysis and isotemporal substitution modeling methods to investigate the association between Chinese middle school students’ 24-hour movement behaviors and the frequency of colds, as well as the impact of reallocating time spent on these behaviors on cold frequency. This not only contributes to understanding the mechanisms through which time-use patterns affect immune health but also provides practical evidence for developing strategies to promote physical activity among adolescents and optimize health interventions. Furthermore, it holds positive significance for advancing interdisciplinary development in the fields of public health and sports science. First, what is the relative distribution of 24-hour movement behaviors and their linear associations with the frequency of colds? This question will be addressed using compositional data regression. Second, how do 15-minute pairwise time re-allocations between different 24-hour movement behaviors (sleep, sedentary behavior, light physical activity, and moderate-to-vigorous physical activity) affect the frequency of colds in middle school students? This question will be addressed using compositional isotemporal substitution modeling. Third, is there a “dose–effect” of isotemporal substitution duration (15/30/45/60-minute re-allocations) on the frequency of colds?

## Participants and Methods

### Participants

The study adopted a cross-sectional design and a random sampling method, selecting students from three middle schools in Wuhan, Hubei Province, China as participants. From grades 7–8 in each school, 3–4 classes were sampled, totaling 469 students who participated in the research. Following the principle of voluntary participation, 438 physically healthy students and their guardians signed the informed consent forms. This study used the ActiGraph GT3X accelerometer to monitor 438 students for one week. Based on the physical activity validity criteria proposed by Anderson, Hagströmer & Yngve (2005), a valid day was defined as wearing the device for at least 10 h, and a valid week required a minimum of three valid days (including two weekdays and one weekend day). Regarding sleep duration assessment, students were instructed to record their daily sleep onset and wake times in a sleep log. Based on the sleep algorithm proposed by Barreria (Barreira et al., 2015), the time points of falling asleep and waking up were manually identified by combining sleep data from behavioral logs, and awake periods during sleep were excluded to calculate the total daily sleep duration. After data screening, results showed that among the 438 participating students, 359 met the accelerometer wear-time criteria (valid rate: 82.0%). Among these 359 students, 356 completed sleep logs were collected. The final valid sample comprised 356 participants. There were 177 boys (49.7%) and 179 girls (50.3%). There were 201 students aged 12 years (56.5%) and 155 aged 13 years (43.5%).

## Methods

### Measurement of physical activity and sedentary behavior

This study was conducted in accordance with the Declaration of Helsinki, and approved by the Central China Normal University (CCNU-IRB-202412012b,12 December 2024). Physical activity and sedentary behavior in the 24-hour period were objectively measured using ActiGraph wGT3X-BT accelerometer with a sampling interval of 30 Hz. Prior to the formal monitoring, the research team conducted preliminary tests on a subset of middle school students to optimize placement of the accelerometer and assess the feasibility of wearing it at least 10 h a day. The validation process for the preliminary test involves testers explaining the purpose and significance of the study to participants, obtaining informed consent, and then guiding them through the test under the supervision of both testers and teachers. After the test, the testers compile and verify the data, and for those with missing data, a retest is conducted or the data is excluded from statistical analysis to ensure data integrity and reliability. Based on the results of the preliminary tests, the researchers provided accelerometer usage guidelines and precautions to the students, parents and teachers. The participants were required to wear the accelerometer on the right hip, removing it only during water-based activities, bathing, and nighttime sleep. After the testing period, the accelerometer data were screened and processed using Actilife 6.13.3 sofware. The cut-points proposed by Evenson et al. (2008) were used to classify the middle school students’ physical activity into different intensity levels. The classification criteria were as follows. Sedentary Behavior (SB), 0–100 counts/min; Light Physical Activity (LPA), 101–2,295 counts/min; Moderate Physical Activity (MPA), 2,296–4,011 counts/min; Vigorous Physical Activity (VPA), ≥4,012 counts/min. Moderate-to-Vigorous Physical Activity (MVPA) was the sum of the MPA and VPA counts/min.

### Measurement of the frequency of colds

The researchers provided the students with a brief description of the clinical symptoms of the common cold, including headache, sneezing, chills, sore throat, runny nose, nasal congestion, coughing, fever, and malaise (Eccles, 2005). This helped students self-diagnose their condition. The frequency of colds in the past year was assessed using the following question: “How many times have you had a cold in the past year?” This question was adapted from similar studies (Matthews et al., 2002; Tang et al., 2024). Respondents were asked to select from the following options: “0 times”, “1 time”, “2 times”, “3 times”, or “≥4 times”. If the frequency of colds exceeded 4 times, the exact number of occurrences was recorded.

### Related control variable investigation

The control variables in this study primarily include gender, age, so-cial economic status (SES) and parental education index. Gender and age were obtained from the class roster of the tested participants, while SES was derived from a questionnaire survey. Parental education index, parental occupation, and household income were used to assess the students’ socioeconomic status. In this study, parental education levels were categorized into three tiers: the starting point was “junior high school or below”, the intermediate point was “high school or vocational secondary school”, and the highest point was “university or above”. Parental occupations were measured with reference to international occupational coding standards. Household income was classified on an annual basis.The final assignment formula adopts the calculation method proposed by Ren (2010) as follows: SES = (0.82  × Parental Education Level + 0.81  × Parental Occupation + 0.76  × Family Income)/0.63.

### Data processing

The data analysis followed the 24-hour movement behavior compositional data analysis guidelines proposed by Chastin et al. (2015) and Dumuid et al. (2018), and was conducted using R statistical software (Version 4.2.3) with relevant compositional data analysis code. (1) First, descriptive statistical analysis was performed on the collected compositional variables, including calculation of compositional geometric means for relevant variables to reflect the time distribution trends of 24-hour movement behaviors. Additionally, variation matrices were used to describe the degree of dispersion in the compositional data. Since the variance of individual data cannot capture the interdependent relationships among activity behaviors, the logarithmic variance of pairwise ratios between all activity behaviors is calculated within compositional data to describe the dispersion trend. Van den Boogaart & Tolosana-Delgado (2013) proposed that the dispersion trend of compositional data is described using a variation matrix, where elements closer to 0 indicate smaller log-ratio variances between the corresponding two components, suggesting stronger co-dependency in the proportions of the components. Additionally, Li et al. (2025) pointed out that a log-ratio variance close to 1 indicates a lower degree of interdependency, while a value close to 0 indicates a higher degree of interdependency. In accordance with the theoretical perspectives of the aforementioned scholars, the analytical methodology is applied to examine the compositional data of the present research. (2) Second, a compositional linear regression model was constructed to examine the association between 24-hour movement behavior compositional data and the frequency of colds. The model employed the isometric log-ratio (ILR) transformation method to address multicollinearity issues among compositional variables, while controlling for gender, age, family socioeconomic status, and parental education index. the ILR transformation for 24-hour movement behaviors was conducted using the ILR function from the compositions package in R software, On this basis, a linear regression model was established: Y_i_=*β*_0_+*β*^T^ilr(x_i_)+*ɛ*_i_. In the model, the *β* values respectively represent the associations between MVPA, LPA, SB, and SP—relative to the other three behaviors—and the frequency of colds, after adjusting for the total time spent on all behaviors and the time spent on each individual other behavior. Whether a model contained errors was determined by examining whether the *P*-values, R^2^, intercept (*β*_0_), and covariate values (*ɛ*_i_) in each model were consistent. (3) Since the dependent variable in this study is the frequency of colds, which represents count data of non-negative integers, Poisson regression is employed to analyze the relationship between the compositional data of 24-hour movement behaviors and the frequency of colds. Poisson regression models event rates and can capture the association between 24-hour movement behaviors and the frequency of cold occurrences under a logarithmic link function. The results are presented in the form of incidence rate ratios (IRR), which carry clear epidemiological interpretation. Additionally, while controlling for demographic variables, this model retains count information and enhances statistical estimation efficiency and interpretative validity. (4) Compositional data isotemporal substitution analysis was employed to examine the impact of “one-to-one” activity substitution on the frequency of colds. Previous studies have indicated that a 15-minute pairwise activity substitution between any two behaviors could lead to significant changes in health outcomes (Dumuid et al., 2018; Fairclough et al., 2017). Therefore, this study used 15-minute isotemporal substitution to analyze the effects of substituting different 24-hour activities on the frequency of colds. (4) Fourth, using 5-minute incremental units, we plotted the predicted changes in the frequency of colds associated with pairwise isotemporal substitutions ranging from −30 to +30 min, to elucidate the “dose–response” relationship between activity redistribution and cold incidence.

## Results

### Distribution characteristics of 24-hour movement behaviors and the frequency of colds among middle school students

Table 1 shows that the compositional geometric means of middle school students’ daily of allocation of time to each physical movement behavior were 560.31 min (38.91%) for SP, 705.23 min (48.97%) for SB, 142.27 min (9.88%) for LPA, and 32.19 min (2.24%) for MVPA. The arithmetic means were 550.91 min (38.26%), 703.82 min (48.88%), 148.72 min (10.33%), and 36.54 min (2.54%), respectively. Discrepancies were observed between the two methods in central tendency. Compared to compositional geometric means, arithmetic means overestimated MVPA and LPA durations, and underestimated SP and SB durations. The distribution pattern based on compositional geometric means met the analytical requirements for compositional data. Additionally, the mean frequency of colds among middle school students within one year was 2.13 ± 2.29. The variance inflation factor (VIF) for all movement behaviors was tested and found to be below 4, with a maximum value of 2.024. This indicates that there is no multicollinearity among the activity behaviors, allowing for further analysis to proceed.

**Table 1:** Geometric means and arithmetic means of the 24-hour movement behaviors in middle school students.

Statistics	SP	SB	LPA	MVPA
Component mean/min	560.31	705.23	142.27	32.19
Component Mean Proportion/%	38.91	48.97	9.88	2.24
Arithmetic mean/min	550.91	703.82	148.72	36.54
Arithmetic Mean Proportion/%	38.26	48.88	10.33	2.54

**Notes.**

SB static behavior LPA low-intensity physical activity MVPA moderate to high-intensity physical activity SP sleep

According to the variation matrix of 24-hour movement behaviors among middle school students (Table 2), the log-ratio variance between SB and SP is the smallest (.0389), indicating the strongest association and lowest mutual substitutability between these two movement behaviors. In contrast, MVPA shows relatively larger log-ratio variances with other behaviors, suggesting weaker associations between MVPA and the other three activity domains. This indicates that time allocation for MVPA is the least stable component within the 24-hour movement behaviors of middle school students.

**Table 2:** Variation matrix of 24-hour movement behaviors in middle school students.

	SP	SB	LPA	MVPA
SP	.0000	.0389	.1216	.2337
SB	.0389	.0000	.1479	.2986
LPA	.1216	.1479	.0000	.1436
MVPA	.2337	.2986	.1436	.0000

**Notes.**

SB static behavior LPA low-intensity physical activity MVPA moderate to high-intensity physical activity SP sleep

### Compositional linear regression analysis of 24-hour movement behaviors and the frequency of colds in middle school students

After adjusting for gender, age, family socioeconomic status, and parental education index, compositional regression analysis was performed with ILR-transformed 24-hour movement behaviors (MVPA, LPA, SB, SP) as independent variables and the frequency of colds as the dependent variable. This allowed us to examine the association between 24-hour time-use composition and cold incidence. Results from the compositional linear regression model (Table 3) revealed a significant negative association between MVPA proportion and the frequency of colds among middle school students (*β* MVPA = −0.91, *P* < .05). This indicates that increased time allocation to MVPA (with corresponding decreases in SB, SP, and LPA proportions) was associated with reduced cold incidence. The proportion of time spent in SP was significantly positively associated with the frequency of colds (*β* SP = 2.56, *p*  < .05). It should be noted that neither SB time proportion (*β* SB = −1.50, *P* > .05) nor LPA proportion (*β* LPA = −1.84, *P* > .05) showed statistically significant associations with the frequency of colds. This indicates that variations in these two behavioral components did not significantly influence the incidence of colds among middle school students.

**Table 3:** Compositional linear regression of 24-hour movement behaviors and the frequency of colds in middle school students.

Movement behaviors	*β*	*P*	Model *P* value	Model *R*^2^
ILR SP/ (SB*LPA*MVPA)	2.56	.00	.57	.04
ILR SB/ (SP*LPA*MVPA)	−1.50	.51		
ILR LPA/ (SP*SB*MVPA)	−1.84	.22		
ILR MVPA/ (SP*SB*LPA)	−0.91	.02		

**Notes.**

*β* values represent the association between relative changes in a given behavior versus all other behaviors and the frequency of colds. For example, ILR [MVPA/(SP⋅ SB⋅ LPA)] indicates the association between changes in MVPA relative to SP, SB, and LPA combined, and the frequency of colds.

### Poisson regression analysis of 24-hour movement behaviors and the frequency of colds among middle school students

Using a Poisson regression model, with controls for variables such as gender, age, family socioeconomic status, and parental education index, this study explored the association between 24-hour movement behaviors and the frequency of colds among middle school students (Table 4). The results showed that the proportion of time spent in MVPA was negatively associated with the frequency of colds (*β* = −0.00077, IRR = .99923, *p* = .054), approaching statistical significance, suggesting that increased MVPA time may be associated with a lower frequency of colds. The proportion of time spent in SP was positively associated with the frequency of colds (*β* = .00021, IRR = 1.00021, *p* = .054), approaching statistical significance, suggesting that an increase in SP time is correlated with a higher frequency of colds. The proportion of time spent in LPA also showed a negative trend (*β* = −0.00024, IRR = .99976, *p* = .100) but did not reach significance. No significant association was found between SB and the frequency of colds (*p* > .05). Among demographic variables, gender had a significant effect the frequency of colds (*β* = .192, IRR = 1.212, *p* = .014), indicating that girls had a significantly higher cold frequency compared to boys. Family socioeconomic status, age, and parental education index showed no statistically significant associations with the frequency of colds (*p* > .05). Overall, the findings suggest that higher levels of MVPA may have a potential protective effect against the frequency of colds in middle school students.

**Table 4:** Poisson regression analysis of 24-hour movement behaviors and the frequency of colds in middle school students.

	*β*	IRR	Std. Err	*Z* value	*P* value
SP	.00021	1.00021	.00011	1.927	.054
SB	−0.00003	.99997	.00005	−0.576	.565
LPA	−0.00024	.99976	.00015	−1.646	.100
MVPA	−0.00077	.99923	.00040	−1.924	.054
gender	.192	1.212	.078	2.459	.014
economy	.114	1.121	.077	1.493	.135
grade	.027	1.028	.076	.357	.721
education	−0.051	.950	.052	−0.982	.326

**Notes.**

IRR is the incidence rate (Incidence Rate Ratio) SB static behavior LPA low-intensity physical activity MVPA moderate to high-intensity physical activity SP sleep

### Predicted changes in the frequency of colds following isotemporal substitution of 24-hour movement behaviors among middle school students

Using 15-minute substitution intervals, this study examined predicted changes in the frequency of colds associated with pairwise substitutions among MVPA, LPA, SB, and SP. The isotemporal substitution analysis (Table 5) revealed that, after adjusting for gender, age, family socioeconomic status, and parental education index, substituting MVPA for SB and SP was associated with significant reductions in the frequency of colds by 0.251 and 0.295 units, respectively. Conversely, substituting SB and SP for MVPA resulted in significant increases of 0.416 and 0.459 units; no other behavioral substitutions showed statistically significant effects.

**Table 5:** Analysis of 15-minute isotemporal substitution among 24-hour movement behaviors and predicted changes in the frequency of colds based on linear regression.

Behavior	Substitute behavior	Frequency of colds (95% CI)
SB	LPA	0.040 (−0.063, 0.144)
SB	MVPA	0.416 (0.029, 0.803)^*^
SB	SP	−0.044 (−0.096, 0.008)
LPA	SB	−0.035 (−0.130, 0.059)
LPA	MVPA	0.381 (−0.073, 0.834)
LPA	SP	−0.080 (−0.178, 0.019)
MVPA	SB	−0.251 (−0.486, −0.017)^*^
MVPA	LPA	−0.211 (−0.522, 0.100)
MVPA	SP	−0.295 (−0.544, −0.046)^*^
SP	SB	0.043 (−0.008, 0.094)
SP	LPA	0.083 (−0.024, 0.190)
SP	MVPA	0.459 (0.058, 0.860)^*^

**Notes.**

SB static behavior LPA low-intensity physical activity MVPA moderate to high-intensity physical activity SP sleep

* *p* < .05.

**Figure 1:**
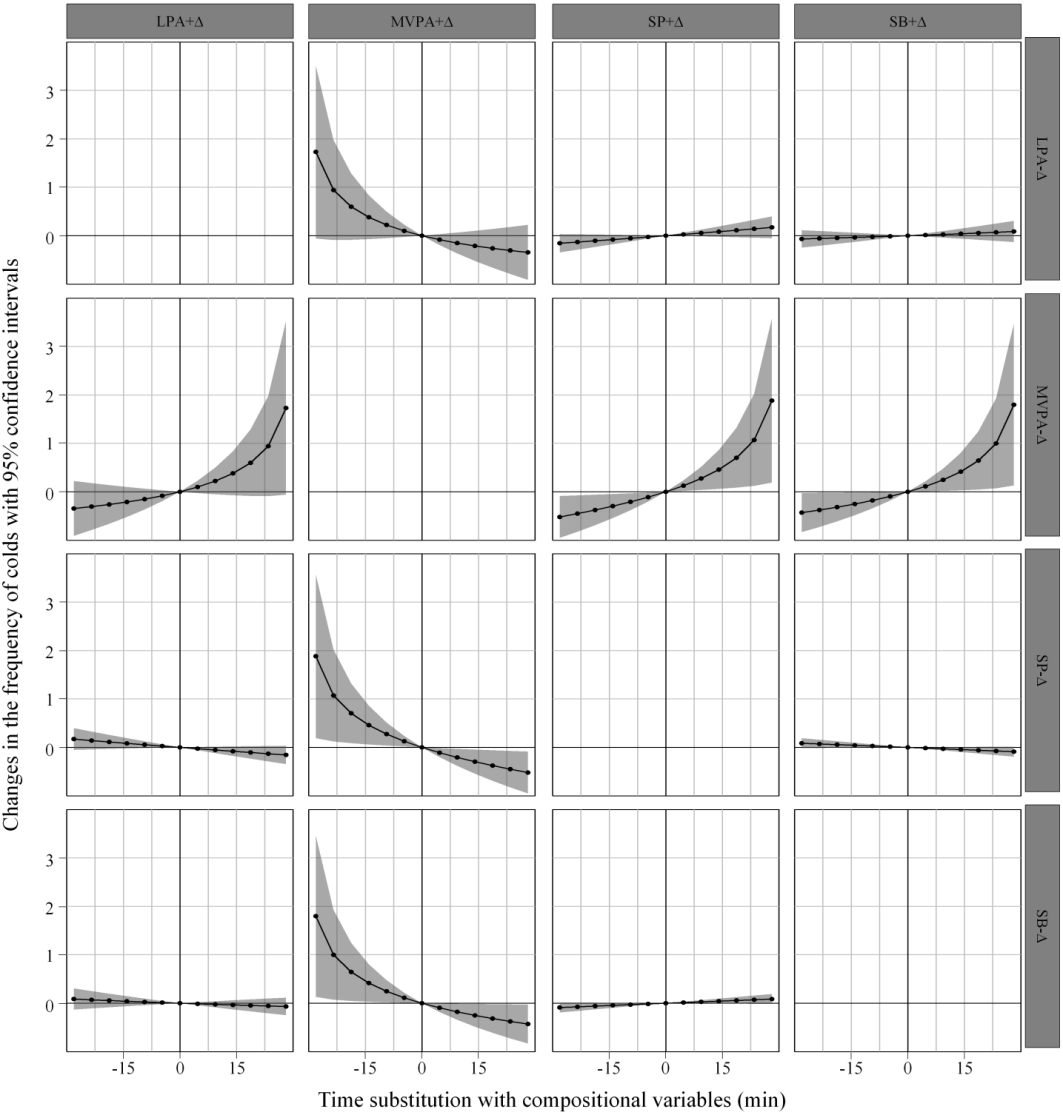
Changing trends in the frequency of colds associated with isotemporal substitution based on linear regression. Note: “+” indicates an increase in the duration of the movement behavior, while “-” denotes a decrease. The shaded areas in the figure represent 95% confidence intervals changes in the frequency of colds. SB, Sedentary Behavior; LPA, Light Physical Activity; MVPA, Moderate-to-Vigorous Physical Activity; SP, Sleep.

Given that the arithmetic mean of MVPA duration was 32.19 min in the study population, we visualized the association trends between pairwise isotemporal substitutions (−30 to +30 min) and the frequency of colds at 5-minute intervals across 24-hour movement behaviors, as presented in Fig. 1. The main findings were as follows. (1) With increasing duration of MVPA substituting for SB and SP time, the total frequency of colds among middle school students demonstrated a sustained downward trend. (2) When substituting 5 min, the total frequency of colds decreased by 0.095 and 0.110 units, respectively. As substitution duration increased from 10 to 30 min, the reductions ranged from 0.178−0.430 to 0.207−0.520 units with gradually diminishing marginal effects. Conversely, increasing substitution of SB and SP for MVPA duration was associated with significant elevations in the frequency of colds. When substituting 5 min, the frequency of colds increased by 0.112 and 0.126 units respectively. As substitution duration extended from 10 to 30 min, the increases ranged from 0.246–1.795 to 0.275–1.882 units, demonstrating significantly accelerated growth rates. Notably, the magnitude of reduction in the frequency of colds induced by MVPA substituting for SB/SP was substantially smaller than the corresponding increase caused by SB/SP substituting for MVPA. Figure 1 illustrates the asymmetric association between these bidirectional activity substitutions and cold incidence among middle school students.

## Discussion

### Analysis of 24-hour movement behaviors and behavioral transitions in middle school students

This study investigated the relative time distribution and transition dynamics of 24-hour movement behaviors among middle school students in Wuhan, China. The results revealed interdependent relationships among these behavioral components. The time allocation proportions for SP, SB, LPA, and MVPA were 560.31 min (38.91%), 705.23 min (48.97%), 142.27 min (9.88%), and 32.19 min (2.24%) respectively. As in previous studies on 24-h movement behaviors in Chinese primary-and middle-school students (Chen et al., 2023), the MVPA proportion had the highest variability, although slight differences were observed in SP, SB, and LPA proportions. Nevertheless, the descending order of daily activity time allocation remained consistent: SB > SP > LPA > MVPA.

The daily sleep duration of the middle school students in this study aligned with the recommendations of the National Sleep Foundation, which suggests 8–10 h per night as the optimal range for adolescents (Robinson et al., 2021). However, the students did not meet the WHO physical activity guidelines for adolescents.Their MVPA was lower than the recommended minimum of 60 min of MVPA per day (Bull et al., 2020). Based on an international systematic review, the mean SB time among Chinese middle school students in this study exceeded the reported range (309–646 minutes/day) observed in children and adolescents from other countries (Pearson et al., 2017). These findings confirm that the middle school students in our sample exhibited prolonged sedentary behavior and insufficient physical activity.

Through an in-depth analysis of the 24-hour movement behaviors change matrix, it was found that the duration of MVPA remains volatile and is the most likely to be substituted by other activities. This may stem from the immense academic pressure faced by middle school students, leaving them with very little discretionary time outside of school. Consequently, MVPA often becomes a “flexible adjustment item” in time allocation—when students need to catch up on sleep due to insufficiency or have excessive homework requiring prolonged sitting, MVPA is the first to be sacrificed. Additionally, the discontinuity of physical education scheduling within the school also makes it difficult for MVPA to occupy a fixed position in daily routines. SB and SP exhibit the lowest mutual substitutability, This may be related to differences in their physiological foundations. SP is rigidly constrained by circadian rhythms and the need for physiological recovery, making it impossible to be genuinely replaced by SB.

### Association between 24-hour movement behaviors and the frequency of colds in middle school students

This study employed isometric log-ratio (ILR) transformation of compositional data to comprehensively examine the association between 24-hour movement behaviors and the frequency of colds. The results revealed a significant negative correlation between MVPA proportion and the frequency of colds, indicating that increased time allocation to MVPA was associated with reduced cold incidence among middle school students. Chubak et al. (2006) conducted a one-year exercise intervention trial and found that moderate-intensity training reduced the incidence of colds in postmenopausal women. Although the methodology differed from the present study, the results were consistent. In the current study, a significant positive correlation was observed between SP proportion in 24-hour movement behaviors and the frequency of colds, indicating that increased sleep duration was associated with higher frequency of colds among middle school students. This study observed a positive correlation between sleep duration and the frequency of colds, which differs from the prevalent conclusion in mainstream research that “sleep duration and physical health exhibit a U-shaped relationship”. The findings may reflect the specific characteristics of the population under investigation: among Chinese adolescents in high-pressure academic environments, excessively long sleep duration often does not represent healthy sleep patterns but may be associated with irregular sleep-wake cycles, insufficient daytime physical activity, or potential suboptimal health status. Specifically, the potential underlying mechanisms may include: the sleep fragmentation and reduced proportion of deep sleep accompanying prolonged sleep, which could weaken the immune-restorative function of sleep; or excessive sleep may displace time for physical activity, leading to decreased overall energy expenditure and inadequate immune function regulation. It is noteworthy that recent research evidence indicates that longer sleep duration (≥9 h per night) may increase the risk of illness (Ayas et al., 2003; Cappuccio et al., 2010). The data from this study show that the average daily SP duration among middle school students reached 560.31 min (over 9 h), which may explain the positive correlation observed between sleep and cold frequency in this population. The findings of this study suggest that when examining the relationship between adolescent sleep duration and health, a comprehensive evaluation framework incorporating “duration, quality, and regularity” should be established, avoiding the simplistic linear equating of sleep duration with health benefits. Future research should utilize longitudinal studies or intervention trials, combined with objective sleep monitoring methods, to further clarify the causal relationship and specific pathways between sleep characteristics and infection risk. Furthermore, neither the proportion of SB nor the proportion of LPA showed statistically significant associations with the frequency of colds in the 24-hour movement behavior composition. As posited by Maher et al. (2014), the health implications of SB and LPA may not operate independently but rather through their displacement effects on MVPA time, thereby indirectly contributing to adverse health outcomes. This study aligns with the findings of Maher et al. (2014) supporting the notion that the impact of movement behaviors on health is holistic and exhibits mutual substitutability. Our data indicate that a combined pattern characterized by higher levels of MVPA and appropriate SP duration is associated with a lower frequency of colds. Therefore, we posit that comprehensive lifestyle interventions aimed at increasing MVPA and optimizing sleep duration may represent a promising approach to reducing the risk of colds among adolescents in the future, though this hypothesis requires validation through longitudinal or experimental studies.

### Association between 24-hour movement behavior reallocation and the frequency of colds in middle school students

This study investigated the associations of time-substitution behaviors in 24-hour activities with the frequency of colds among middle school students. Isotemporal substitution analysis revealed that mutual substitution between MVPA and both SB and SP was significantly associated with the frequency of colds. On average, replacing 15 min per day of SB or SP with an equivalent duration of MVPA could reduce the frequency of colds. By comparing changes in the frequency of colds after time reallocation, this study found that redistributing time from SB and SP to MVPA significantly reduced the total frequency of colds among middle school students.

The results demonstrate that increasing MVPA time may be the optimal approach to lowering the frequency of colds in this population. From a physiological perspective, MVPA enhances immune function by stimulating blood circulation and promoting the continuous exchange of white blood cells between tissues. This process increases the activity of key immune cells, including neutrophils, natural killer (NK) cells, and cytotoxic T cells, while also boosting the secretion of immunoglobulin A (IgA) in saliva. The exercise-induced elevation of these anti-pathogenic cells strengthens the body’s immune surveillance and reduces the risk of infections such as the common cold (Adams et al., 2011; Sellami et al., 2018). However, the isotemporal substitution of MVPA for LPA did not yield significant effects. This may be attributed to the fact that both MVPA and LPA fall within the physical activity domain, and while they exhibit intensity-gradient differences in metabolic regulation, their marginal effects are likely smaller compared to substituting SB or SP.

This study further investigated the dose–response relationships between MVPA and SB/SP/LPA. The findings revealed an asymmetric dose–response pattern in the isotemporal substitution effects between MVPA and both SB and SP on cold frequency. This observed asymmetry may stem from two key factors. First, there were proportional time allocation differences in our sample. In this study, 15 min of MVPA accounted for 46.6% of the average daily MVPA duration (32.19 min), whereas equivalent 15-minute substitutions represented only 2.7% of total SP time and 2.1% of SB time. This substantial disparity in relative time proportions likely contributed to the unequal substitution effects. Second, there are cumulative health benefits derived from physical activity in the general population, but discontinuation or abandonment of regular physical activity leads to rapid deterioration of these benefits. Specifically, when individuals persist in physical activity, especially increasing MVPA activity time, the risk of diseases such as colds will be reduced; when individuals stop physical activity and reduce MVPA activity time, the negative impact on colds will be greater.

## Conclusions

This study systematically examined the association between 24-hour movement behaviors and the frequency of colds among Chinese middle school students. The study results indicate that the proportion of time spent on MVPA is significantly negatively correlated with the frequency of colds among middle school students, while the proportions of time spent on LPA and SB show no statistically significant association with cold frequency. Isotemporal substitution of MVPA for SB and SP leads to significant changes in the frequency of colds among middle school students. Specifically, as the duration of MVPA replacing SB and SP increases, the frequency of colds significantly decreases, and conversely, it increases. Furthermore, the isotemporal substitution effects of MVPA for SB and SP are asymmetric.

This study has certain limitations. First, as a cross-sectional survey, the design of this study inherently precludes inferring causal relationships between different movement behaviors and the frequency of colds. Future research could employ intervention trials or longitudinal tracking designs to examine whether causal links exist between the substitutive effects of movement behaviors and cold frequency. In light of the finding in this study that sleep duration proportion is positively associated with cold frequency, attention to adolescent sleep health should extend beyond a singular focus on “duration” and instead establish a comprehensive evaluation framework encompassing “duration, quality, and regularity”. This approach avoids equating sleep duration linearly with health benefits in a simplistic manner. To establish causal relationships, future studies need to adopt longitudinal designs or intervention trials, combined with objective monitoring tools such as actigraphy, to further clarify the causal pathways and specific mechanisms linking different movement behaviors—particularly complex sleep characteristics—to infection risk. Second, the data on the frequency of colds in this study relied on self-reports from middle school students regarding the past 12 months, which may introduce recall bias. Considering that adolescents may forget or misjudge mild cold episodes of short duration, this bias is more likely to result in a systematic underestimation of the incidence of colds, potentially weakening the observed effect size of the association between 24-hour movement patterns and the frequency of colds in this study. Future research could mitigate the impact of recall bias on effect estimation by incorporating medical records, parent-assisted reports, or employing dynamic monitoring methods such as prospective health diaries and regular short-term recalls (*e.g.*, monthly) to more accurately capture cold incidents. Third, since this study did not measure or control for potential confounding variables such as seasonal factors (*e.g.*, peak cold seasons), individual vaccination status, and environmental factors (*e.g.*, regional air quality index), the results may have been influenced by these omissions. Specifically, unaccounted seasonal fluctuations could confound the temporal relationship between behavioral patterns and the frequency of colds; unrecorded variations in vaccination status may partly explain differences in individual susceptibility to infections; and overlooking long-term or short-term environmental pollution exposure could obscure the interactive effects of environment and behavior on respiratory health. Together, these factors limit the precision of this study’s inference regarding the independent association between “behavior and colds”. Future research should systematically collect data on these covariates during the design phase—for instance, by incorporating time variables, linking to regional environmental monitoring databases, and using questionnaires to assess vaccination history. These variables should then be included in statistical models as covariates or stratification variables to more accurately estimate the independent impact of 24-hour movement behaviors on cold risk. Fourth, During the implementation of this study, the failure to exclude students experiencing active cold symptoms may have introduced reverse causality interference due to illness-induced secondary behavioral changes (*e.g.*, increased sleep due to fatigue or reduced physical activity due to discomfort). Specifically, these short-term, pathology-driven behavioral adjustments could confound and amplify the association between the “24-hour movment behavior patterns” and “ the frequency of colds” investigated in this study, particularly potentially overestimating the positive relationship between sleep duration and the frequency of colds. To enhance the clarity of causal inference, future research could implement health screening during baseline assessments to exclude individuals in the phase of a cold infection, or adopt a prospective design to commence behavioral monitoring and follow-up only after confirming participants’ healthy status. Additionally, sensitivity analyses could be conducted during data analysis to examine whether excluding cases of cold onset shortly after enrollment alters the main findings, thereby evaluating the potential impact of this confounding factor on the robustness of the conclusions.

Based on the research findings, we recommend that schools and families strengthen collaboration to jointly optimize the 24-hour activity patterns of middle school students, thereby fostering the comprehensive development of adolescent physical and mental health through scientific time allocation. Specifically, at the school level, it is advisable to explore optimizing curriculum structures, enhancing the activity intensity of physical education classes, recess breaks, and after-school extended services, while ensuring the completion of teaching tasks, to create more opportunities for students to engage in moderate-to-vigorous physical activity. At the family level, emphasis should be placed on reasonably arranging after-school time, reducing prolonged sedentary behaviors—particularly unnecessary recreational screen time—to promote healthier time allocation between physical activity and static behaviors.

##  Supplemental Information

10.7717/peerj.21124/supp-1 Supplemental Information 1 Raw data details

10.7717/peerj.21124/supp-2 Supplemental Information 2 STROBE checklist

10.7717/peerj.21124/supp-3 Supplemental Information 3 Raw data

## Additional Information and Declarations

### Data Availability

The following information was supplied regarding data availability: The raw data are available in the Supplemental File.
