# Supplementary material for: Association between 24-hour movement behaviors and the frequency of colds in Chinese middle school students: a compositional and isotemporal substitution analysis
Source: PeerJ. 2026 Apr 24;14:e21124. doi: 10.7717/peerj.21124 (PMC13116403; doi:10.7717/peerj.21124)
Supplement: Supplemental Information 1 [file peerj-14-21124-s001.doc]

**Raw data description**

SP：Sleep

SB：Sedentary Behaviour

LPA：Light-Intensity Physical Activity

MVPA：Moderate-to-Vigorous Physical Activity

y：Frequency of cold

Among them, MVPA, LPA, SP, and SB represent the duration of activities in a week, with the unit being minutes; in "gender", 1 stands for male students and 2 for female students; in "economy", 1 indicates a relatively better family economic status and 2 a relatively poorer one; in "grade", 1 refers to 7th-grade students and 2 to 8th-grade students; in "education", 1 represents a relatively better parental education level and 2 a relatively poorer one.

Under the unique cultural context of China, academic burden is a key factor influencing the 24-hour activity behaviors (sleep, physical activity, sedentary behavior) of adolescents, which may further affect their health outcomes. To address the current research gap in applying compositional data analysis to explore the association between activity behaviors and frequency of colds among Chinese middle school students, this study collected the following data. This dataset provides a foundation for the first research applying this methodological framework to investigate the relationship between time allocation and health.

1.Data Source: This study employed a cross-sectional design and selected students from three middle schools in Wuhan City, Hubei Province, China, through random sampling. From each school, 3–4 classes were randomly chosen from grades 7 to 8, resulting in a total of 469 students participating in the study. Adhering to the principle of voluntary participation, 438 physically healthy students and their guardians signed informed consent forms. The study used ActiGraph GT3X accelerometers to monitor the 438 students for one week. Data screening showed that among the 438 participants, 359 met the accelerometer wear-time criteria (valid rate: 82.0%). Among them, 356 students completed sleep logs, yielding a final valid sample of 356 participants. The gender distribution was 177 males (49.7%) and 179 females (50.3%), while the age distribution was 201 participants aged 12 (56.5%) and 155 participants aged 13 (43.5%).

2.Main Variables: The dependent variable of this study is the frequency of colds（Y）, operationally defined as the number of self-reported colds by students in the past year. The core independent variable is the 24-hour movement behavior composition, which consists of four components: sleep time（SP）, sedentary behavior time（SB）, light-intensity physical activity time（LPA）, and moderate-to-vigorous physical activity time（MVPA）. Among these, sedentary behavior and all intensity levels of physical activity were objectively measured using the ActiGraph GT3X+ accelerometer, while sleep time was obtained from sleep logs completed by the students. These four components are mutually exclusive and sum to 1440 minutes per day. Control variables primarily include gender, economy, grade, and education.

3.Data Processing: ActiLife 6.13.3 software was used for data screening and processing, while R 4.2.3 statistical software and related compositional data analysis code packages were employed for statistical analysis.
